# Eye Comfort and Physiological Reconstruction of an Entire Upper Eyelid Defect

**Published:** 2020-05-29

**Authors:** Yasuhiro Sakata, Katsuya Okuda, Yoshitaka Wada, Shinji Kumegawa, Hirohisa Kusuhara, Noritaka Isogai, Shinichi Asamura

**Affiliations:** ^a^Departments of Plastic and Reconstructive Surgery; ^b^Otolaryngology-Head and Neck Surgery, Wakayama Medical University, Wakayama City, Wakayama, Japan; ^c^Department of Plastic and Reconstructive Surgery, Faculty of Medicine, Kindai University, Osaka City, Osaka, Japan

**Keywords:** reconstruction, upper eyelid, Merkel cell carcinoma, forearm flap, free flap

## Abstract

Objective: Reconstruction of an extensive full-thickness upper eyelid defect is challenging. The purpose of this report is to introduce this procedure with emphasis on reconstruction of the eyelid margin to obtain eye comfort. Methods: We designed a technique using a radial forearm flap for the outer layer to reconstruct the entire eyelid after resection of Merkel cell carcinoma. In additional, the inner layer and the eyelid margin were reconstructed with a buccal mucosal graft and a reverse Hughes flap. Results: There has been no recurrence of the tumor, opening and closing functions of the eyelid are maintained, and the patient has not complained of eye discomfort. Conclusion: Maintenance of mobility, flexibility, and a good ocular surface in contact with the sensitive cornea are the main foci of upper eyelid reconstruction, with an optimal fissure height and an appropriate contour of the eyelid. In addition, to obtain eye comfort, it is important to protect the cornea without significantly restricting eyelid mobility.

Reconstruction of the entire upper eyelid is a challenging procedure for plastic surgeons. Even if functional repair is perfect, patients may not be satisfied with the cosmetic results, or vice versa. Total reconstruction of the upper eyelid can be performed with lower eyelid tissue using the Cutler-Beard procedure or the Mustard method^1^^-^3; however, it is difficult to reconstruct wide defects that extend beyond the medial or lateral canthus using these methods. In the case reported here, total upper eyelid reconstruction was performed using a radial forearm flap for the outer layer and a buccal mucosal graft and a reverse Hughes flap for the inner layer,^4^ which contains the eyelid margin. The purpose of this report is to introduce this procedure with an emphasis on reconstruction of the eyelid margin to obtain eye comfort.

## CASE REPORT

**Figure 1:**
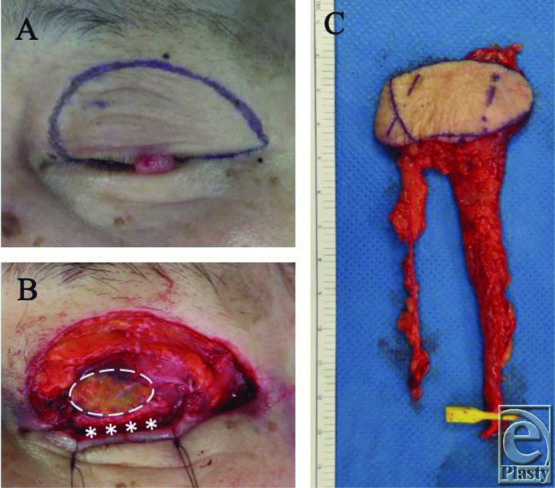
(*a*) Tumor resection with 20-mm margins. (*b*) The inner layer and the eyelid margin were reconstructed with a buccal mucosal graft (dotted line) and a reverse Hughes flap (*). (*c*) A radial forearm flap was harvested.

A 76-year-old woman had Merkel cell carcinoma of the left upper eyelid. The tumor was about 7 × 4 mm and was present in the lateral upper eyelid margin. Tumor resection with 20-mm margins including the levator muscle and septum caused a full-thickness upper eyelid defect of about 60 × 30 mm (Fig 1*a*).

The inner layer and the eyelid margin were reconstructed with a buccal mucosal graft and a reverse Hughes flap (tarsoconjunctival flap from the lower eyelid).^4^ The lower eyelid was everted and the tarsoconjunctival flap was transposed from the lower to upper eyelid margin. To create the flap, a horizontal incision was made through the conjunctiva and tarsus 2 mm below the eyelid margin and a downward vertical incision was made. The pedicle tarsoconjunctival flap was mined between the conjunctiva and eyelid retractors and advanced to the upper eyelid defect. The flap was then sutured with 7-0 Vicryl sutures medially and laterally (Fig 1*b*). Stage 2 takedown of the pedicle flap was performed by easy cutting of the conjunctiva along the new upper eyelid margin with scissors after 2 weeks. An 80 × 40-mm radial forearm flap was harvested (Fig 1*c*). Flap vessels were anastomosed to the superficial temporal artery and vein and then the outer layer was reconstructed with the flap.

## RESULT

**Figure 2:**
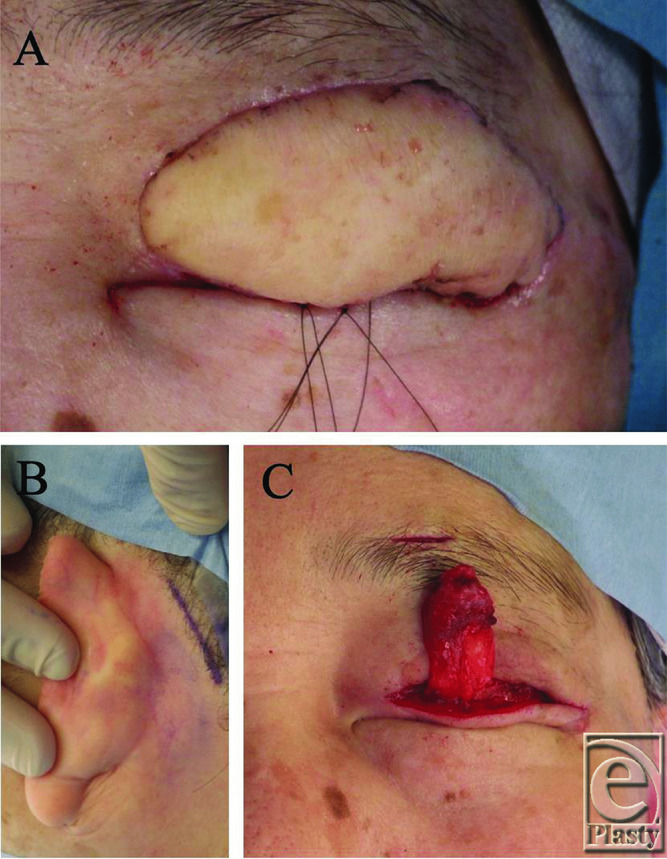
(*a*) The patient underwent simultaneous weight loss surgery and frontalis suspension surgery with a postauricular fascia graft. (*b*) The fascia was harvested after a skin incision along the edge of the hairline of the postauricular region. (*c*) The postauricular fascia was fixed at the subcutaneous tissue of the brow.

About 6 months later, the patient underwent simultaneous weight loss surgery and frontalis suspension surgery with a postauricular fascia graft (Fig 2*a*). The fascia was harvested after a skin incision along the edge of the hairline of the postauricular region (Fig 2*b*). This incision was made 6 mm from the eyelid margin, and the central area of the upper brow margin was also incised. A tunnel was made from the brow incision through the subcutaneous tissue layer and reached up to the tarsal plate. The fascia was sutured with 6-0 nylon on the tarsal plate and then the upper eyelid curvature was confirmed by pulling the fascia through the subcutaneous tissue tunnel. After the upper eyelid height was adjusted appropriately with a trial suture at the brow incision, the fascia was fixed at the subcutaneous tissue of the brow (Fig 2*c*).

**Figure 3:**
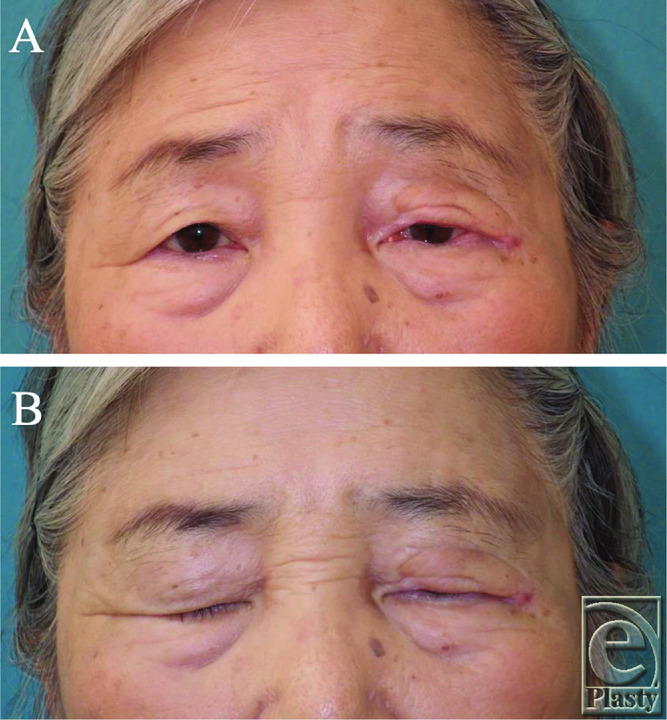
Three years after surgery: Postoperative eyelid opening (*a*) and eyelid closing (*b*). The patient had not complained of any eye discomfort.

Three years after the first surgery, there had been no recurrence of the tumor, opening and closing functions of the eyelid were maintained, and the patient had not complained of any eye discomfort (Figs 3*a* and 3*b*).

## DISCUSSION

A malignant tumor in the palpebral region should be resected with a large oncological safety margin. During resection of Merkel cell carcinoma, a distance of 2 cm from the cancer-free border is recommended.^5^ As a result, all layers of the upper eyelid are lost after resection and reconstruction of the total upper eyelid defect is needed.^1^^-^3 The type of reconstruction depends on anatomic deficits, including the vertical, horizontal, and depth dimensions and availability of regional and distant tissues for reconstruction. There are limitations to the size of an upper eyelid defect that can be covered by tissue from the local palpebral or lateral orbit region.^1^^-^3^,^^6^ Thus, in such cases, reconstruction with a free flap is required for the outer layer with the skin.

Microsurgical techniques have revolutionized postablative reconstruction. The palmaris longus tendon is useful in supporting the radial forearm flap in head and neck reconstruction,^7^ and Iwanaga et al^8^ described a suspension technique that involved securing the palmaris longus tendon of the forearm flap to the frontalis muscle in entire upper eyelid reconstruction. Our case was exceptional in that the patient who was one of about 5% of Japanese people who have palmaris longus tendon defects.^9^ Therefore, frontalis suspension could not be performed in the first surgery and was performed in a second surgery.

Anatomically, the eyelid contains 3 major layers: the outer layer with skin, the inner layer with mucosa, and the semi-rigid tissue “tarsal plate” between these 2 layers. Inability to close the eyelids perfectly can cause drying of the eye, which leads to blurred vision, light sensitivity, and an increased risk of infection that could eventually lead to loss of the eye. To avoid reducing the function of the eyelids, transplantation of semi-rigid tissue is required and reconstruction of the eyelid margin is extremely important.

The original Hughes procedure was described by Hughes^10^ about 80 years ago with regard to the inner layer and the eyelid margin for lower eyelid reconstruction. The original procedure used the tarsal plate and the conjunctiva, as well as the Muller and levator muscles, as a transconjunctival flap. Over the years, the Hughes procedure has been improved to allow use of the tarsal plate and conjunctiva alone. As a method for the inner layer and the eyelid margin for upper eyelid reconstruction, the standard reverse modified Hughes procedure was described in 1994. This procedure has the advantage of using the same eyelid tissue for reconstruction instead of tissue from other body sites. Physiologically, this is an ideal approach to avoid ocular complications.

To data, only hard palate and buccal mucosa have been used to reconstruct the inner layer and reconstruction of the eyelid margin has not been performed. As a result, corneal disorder is prolonged and eye comfort cannot be acquired after the operation. In previous reports of free flap reconstruction, patients did not have corneal pain or need eye drops in the long term, but there was no description of the length of symptoms and use of eye drops after surgery. Since our patient complained of eye discomfort, we prescribed eye drops for 2 months after the frontalis suspension procedure, but eye comfort was achieved without eye drops thereafter.

Reconstruction of the upper eyelid must also result in internal means of movement to raise the eyelid above the pupillary area for vision and to close the eyelid reflexively for lubrication of the cornea and protection from injury. The forearm flap is often flexible and thin; therefore, it is usually ideal for reconstruction of the outer layer of the entire upper eyelid. The semi-rigid “tarsal plate” tissue is not only a fixed source of graft material used in the frontalis suspension procedure but also a physiologically necessary tissue. It should have an optimal fissure height and an appropriate contour of the eyelid since maintenance of the corneal surface is critical for sharp vision and patient comfort.
